# Supplementary material for: Functional composition has stronger impact than species richness on carbon gain and allocation in experimental grasslands
Source: PLoS One. 2019 Jan 31;14(1):e0204715. doi: 10.1371/journal.pone.0204715 (PMC6354960; doi:10.1371/journal.pone.0204715)

S1 Fig. Average air δ^{13}C-CO_2 signature during the pre-enrichment phase (a) and daily means of δ^{13}C-CO_2 at the outlet of the experimental units based on measurements every two hours during the period of continuous atmospheric $^{13}CO_2$ labelling (b). Shown are means (± 1 SD) for experimental units representing mixtures with 4 and 16 sown species, respectively, and values of ambient control measured in the common air conduit before entering the experimental units. Note the different range of the y-axes in the two graphs.

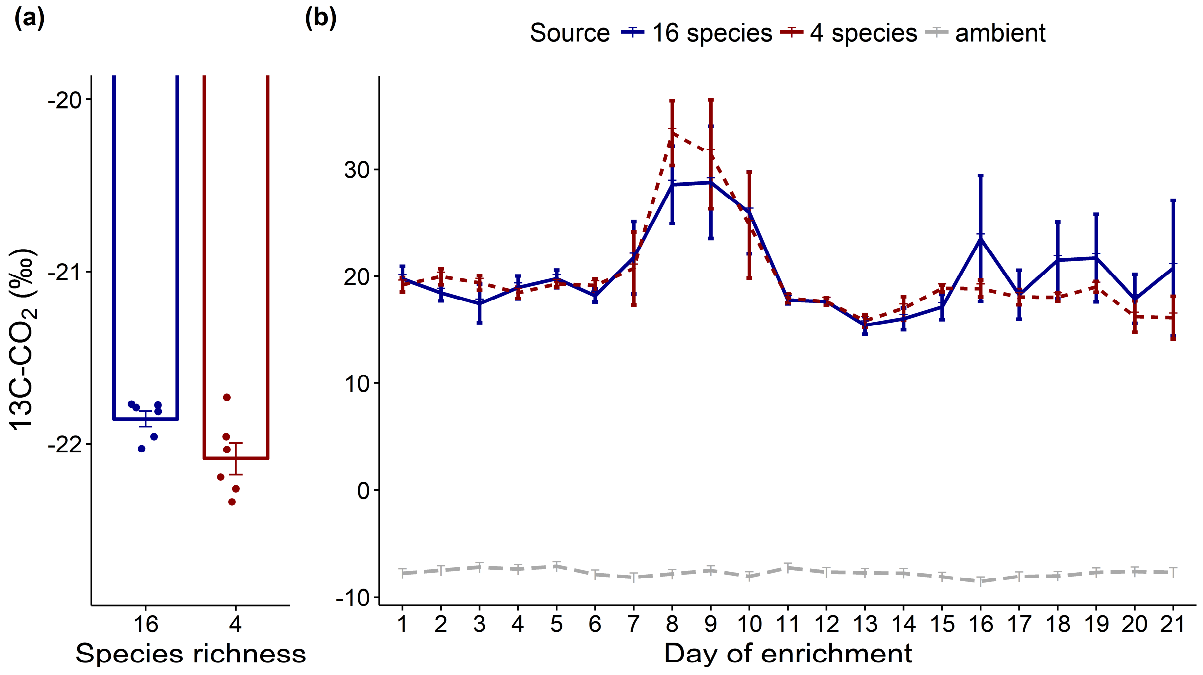

Supplement: S1 Fig — (PDF) [file pone.0204715.s001.pdf]
