# Supplementary material for: Functional composition has stronger impact than species richness on carbon gain and allocation in experimental grasslands
Source: PLoS One. 2019 Jan 31;14(1):e0204715. doi: 10.1371/journal.pone.0204715 (PMC6354960; doi:10.1371/journal.pone.0204715)

S2 Fig. Scatterplots between species-level shoot ^{13}C abundance and functional traits. Shown are species-level shoot ^{13}C abundance and (a) leaf nitrogen concentration, (b) stomatal conductance, (c) leaf greenness, and (d) shoot height. Different symbols indicate values of species assigned to different functional groups.

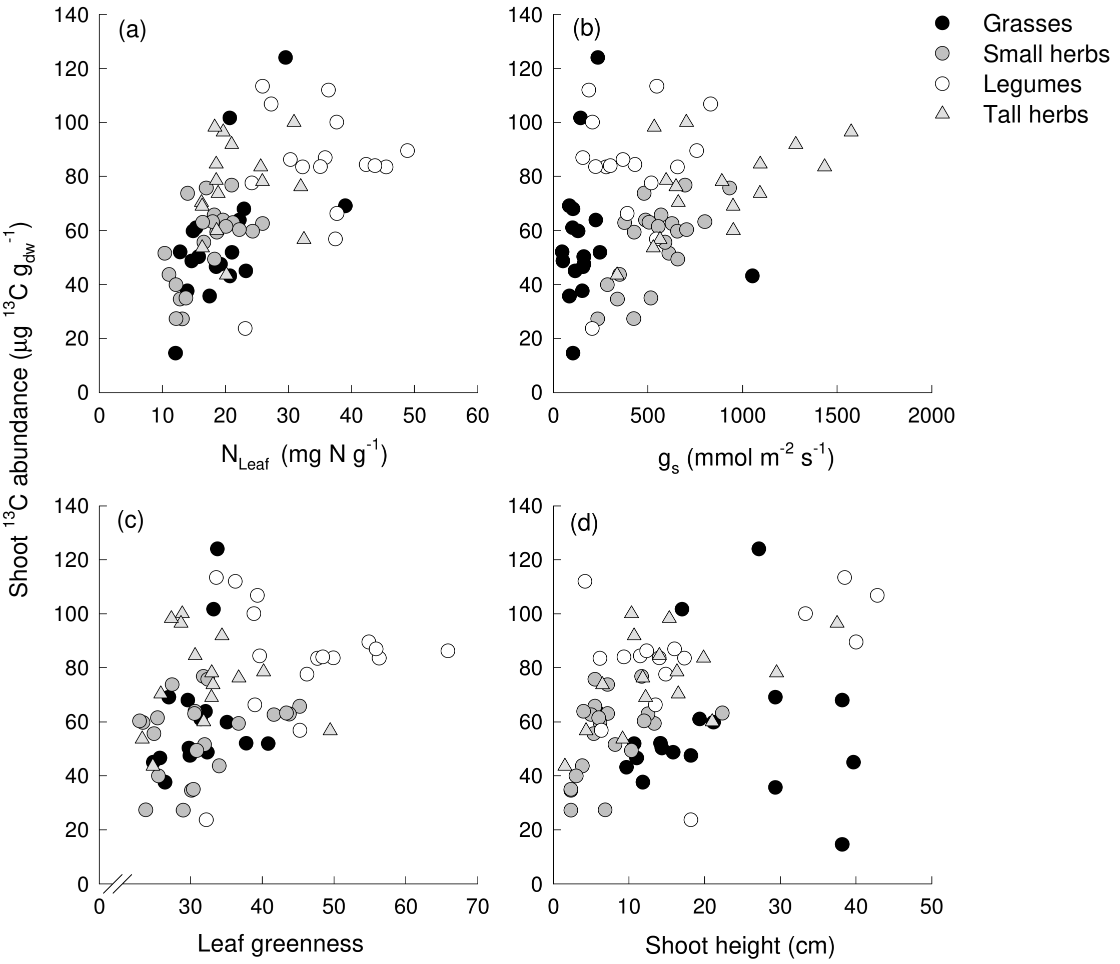

Supplement: S2 Fig — (PDF) [file pone.0204715.s002.pdf]
