# Supplementary material for: Functional composition has stronger impact than species richness on carbon gain and allocation in experimental grasslands
Source: PLoS One. 2019 Jan 31;14(1):e0204715. doi: 10.1371/journal.pone.0204715 (PMC6354960; doi:10.1371/journal.pone.0204715)

S3 Fig. Non-structural carbohydrate concentrations and $\delta^{13}C$ in relation to functional groups. (a-e) ^{13}C abundance, (f-k) ^{13}C atom% excess, and (l-p) concentrations in leaves for (a, f, l) glucose, (b, g, m) fructose, (c, h, n) sucrose, (d, i, o) RFO, and (e, k, p) starch. Note that data are identical with those in Fig 5, but are presented with a focus on functional group differences. Shown are means (+1 SE) per functional group averaged across all mixtures. Results of Tukey`s test ($P < 0.05$) applied to test for differences among functional groups are indicated with letters. Abbreviations are: GR = grasses, SH = small herbs, TH = tall herbs, LE = legumes.

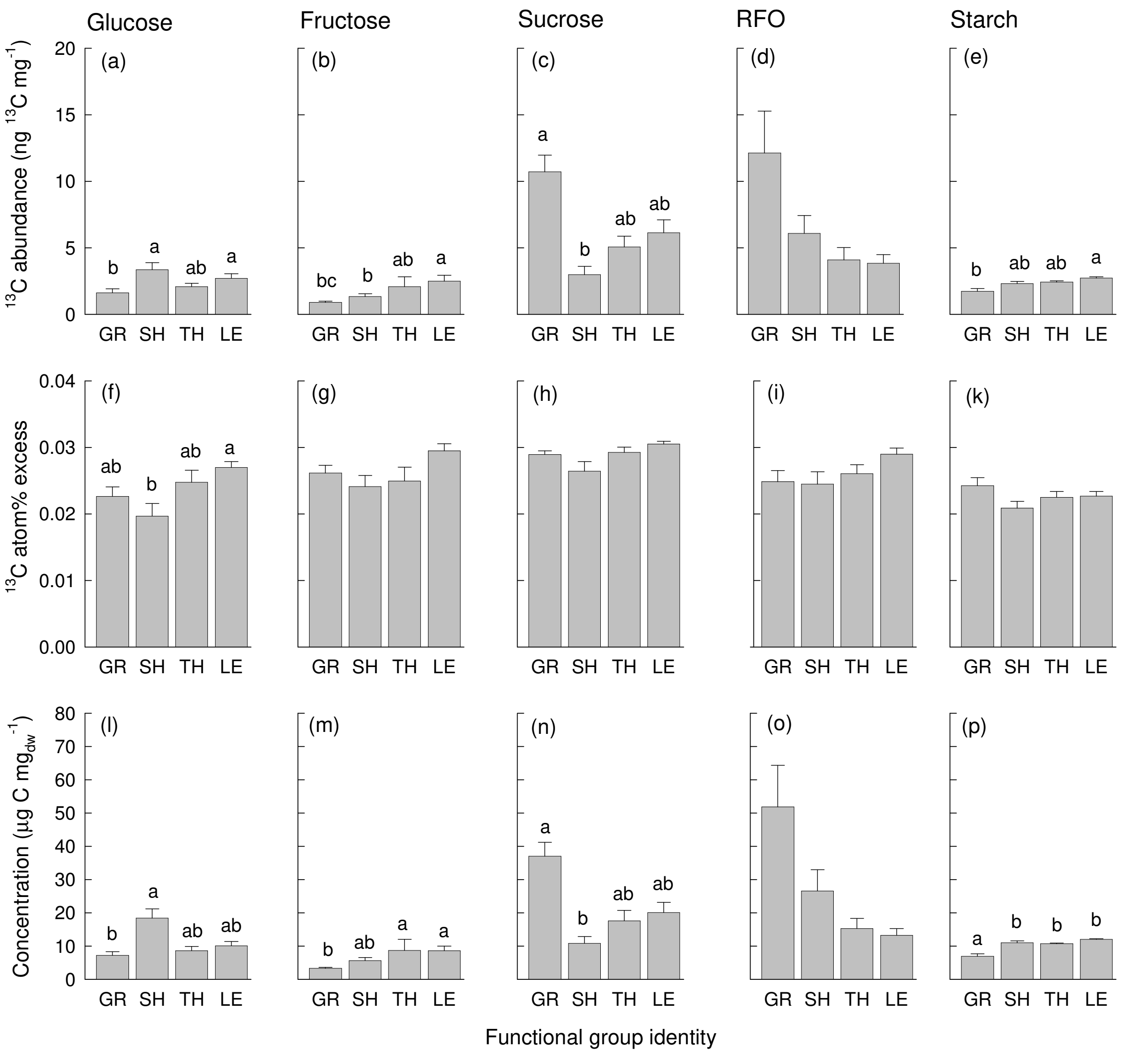

Supplement: S3 Fig — (PDF) [file pone.0204715.s003.pdf]
