# Supplementary material for: Functional composition has stronger impact than species richness on carbon gain and allocation in experimental grasslands
Source: PLoS One. 2019 Jan 31;14(1):e0204715. doi: 10.1371/journal.pone.0204715 (PMC6354960; doi:10.1371/journal.pone.0204715)
Supplement: S1 Method — (PDF) [file pone.0204715.s004.pdf]

S1 Method. Measurement of aboveground functional traits.

Aboveground plant functional traits expected to be relevant for carbon uptake and metabolism were measured for all available plant species in each experimental unit from 17-19 July 2012. Stomatal conductance and leaf greenness were measured for three fully-expanded leaves per species in each experimental unit. Stomatal conductance (g_s; mmol m^{-2} s^{-1}) was measured with a leaf porometer (Decagon Devices, Inc.) using the auto mode of the porometer (taking the first 30 s of stomatal conductance data to predict the final stomatal conductance occurring under true steady state conditions). Leaf greenness (LeafG; unitless), an estimate of chlorophyll concentrations, was obtained by measuring the absorption of two different wavelength (650 nm and 940 nm) with a portable chlorophyll meter (SPAD-502; Konica-Minolta, Osaka, Japan). The growth height of three shoots (H_{Shoot}; cm) per species and unit was measured with a ruler. Afterwards, bulk samples of 3 to 9 fully-expanded mature leaves per species and unit were collected and put into moistened paper towel in sealed plastic bags for re-hydration and stored overnight at 4°C. Then, leaf samples were blotted dry with tissue paper to remove any surface water and weighed immediately to determine water-saturated fresh weight. Leaf area was measured with a leaf area meter (LI-3100, LI-COR, USA). Samples were oven-dried (65°C, 48h) and weighed. Leaf dry matter content (= dry mass per unit fresh mass, LDMC; mg g^{-1}) and specific leaf area (= leaf area per unit dry mass, SLA; mm^2 mg^{-1}) were calculated from these samples. Leaf samples were milled to a fine powder and analysed with an elemental analyzer (Flash EA 1112, Thermo Italy, Rhodano, Italy) to obtain leaf N concentrations (N_{leaf}; mg N g^{-1}).
