# Supplementary material for: Functional composition has stronger impact than species richness on carbon gain and allocation in experimental grasslands
Source: PLoS One. 2019 Jan 31;14(1):e0204715. doi: 10.1371/journal.pone.0204715 (PMC6354960; doi:10.1371/journal.pone.0204715)
Supplement: S1 Table — (PDF) [file pone.0204715.s005.pdf]

S1 Table. Species composition of studied mixtures.

Dome	SR	Species composition
D02	4	(GR) **<u>ArrEla</u>**, (SH) **<u>PlaLan</u>**, (TH) AntSyl, (LE) TriCam
D04	4	(GR) **<u>FesPra</u>**, (SH) **<u>PlaLan</u>**, (TH) CamPat, (LE) OnoVic
D05	4	(GR) BroHor, (SH) **<u>RanRep</u>**, (TH) LeuVul, (LE) **TriFra**
D08	4	(GR) **<u>AntOdo</u>**, (SH) **<u>PruVul</u>**, (TH) **KnaArv**, (LE) TriPra
D10	4	(SH) **<u>PlaMed</u>**, ScoAut, (TH) **KnaArv**, (LE) **<u>VicCra</u>**
D12	4	(GR) **<u>TriFla</u>**, (TH) **HerSph**, TraPra, (LE) **<u>MedVar</u>**
D01	16	(GR) CynCri, **<u>FesPra</u>**, **PhlPra**, **PoaTri**, **<u>TriFla</u>**, (TH) **<u>AchMil</u>**, CamPat, **CenJac**, **RumAce**, SanOff, (LE) **<u>LatPra</u>**, LotCor, **OnoVic**, TriHyb, **TriRep**, **VicCra**
D03	16	(GR) AntOdo, BroHor, **<u>HelPub</u>**, PoaPra, (SH) **AjuRep**, **<u>PlaLan</u>**, **RanRep**, **<u>TarOff</u>**, (TH) AntSyl, CarCar, **<u>GerPra</u>**, TraPra, (LE) **LatPra**, LotCor, TriCam, VicCra
D06	16	(GR) **AloPra**, BroHor, CynCri, **PoaPra**, (SH) **AjuRep**, **<u>PlaMed</u>**, PriVer, RanRep, (TH) AntSyl, **CamPat**, CarPra, **<u>GerPra</u>**, (LE) MedLup, TriCam, **TriDub**, TriRep
D07	16	(GR) **<u>AloPra</u>**, BroHor, CynCri, LuzCam, (SH) **<u>PlaMed</u>**, ScoAut, **TarOff**, **<u>VerCha</u>**, (TH) CarCar, **CreBie**, **HerSph**, **<u>PimMaj</u>**, (LE) **<u>LatPra</u>**, **OnoVic**, TriCam, TriHyb
D09	16	(GR) AntOdo, **BroEre**, **FesRub**, PhlPra, (SH) **AjuRep**, **<u>BelPer</u>**, **RanRep**, **<u>VerCha</u>**, (TH) **<u>CreBie</u>**, **GalAlb**, **GerPra**, **RumAce**, (LE) **OnoVic**, TriDub, TriFra, **VicCra**
D11	16	(GR) AntOdo, **<u>ArrEla</u>**, BroHor, **<u>FesPra</u>**, **<u>HelPub</u>**, **PoaTri**, (SH) **<u>AjuRep</u>**, **<u>GleHed</u>**, PruVul, **RanRep**, **TarOff**, (LE) LotCor, **<u>MedVar</u>**, **TriPra**, TriRep, **<u>VicCra</u>**

Dome is the experimental unit in the Ecotron experiment. SR indicates the sown species richness-level. Species analysed for ^{13}C abundance in shoot biomass are highlighted and species included in analyses of non-structural carbohydrates are additionally underlined.

Abbreviations of species names:

Grasses (GR): AloPra: *Alopecurus pratensis* L.; AntOdo: *Anthoxanthum odoratum* L.; ArrEla: *Arrhenatherum elatius* (L.) J. et C. Presl; BroEre: *Bromus erectus* Huds.; BroHor: *Bromus hordeaceus* L.; CynCri: *Cynosurus cristatus* L.; FesPra: *Festuca pratensis* Huds.; FesRub: *Festuca rubra* L.; HelPub: *Helictotrichon pubescens* (Huds.) Pilg.; LuzCam: *Luzula campestris* (L.) Dc.; PhlPra: *Phleum pratense* L.; PoaPra: *Poa pratensis* L.; PoaTri: *Poa trivialis* L.; TriFla: *Trisetum flavescens* (L.) P. Beauv.

Small herbs (SH): AjuRep: *Ajuga reptans* L.; BelPer: *Bellis perennis* L.; GleHed: *Glechoma hederacea* L.; PlaLan: *Plantago lanceolata* L.; PlaMed: *Plantago media* L.; PriVer: *Primula veris* L.; PruVul: *Prunella vulgaris* L.; RanRep: *Ranunculus repens* L.; ScoAut: *Scorzoneroides autumnalis* (L.) Moench; TarOff: *Taraxacum officinale* Wiggers; VerCha: *Veronica chamaedrys* L.

Tall herbs (TH): AchMil: *Achillea millefolium* L.; AntSyl: *Anthriscus sylvestris* (L.) Hoffm.; CamPat: *Campanula patula* L.; CarPra: *Cardamine pratensis* L.; CarCar: *Carum carvi* L.; CenJac: *Centaurea jacea* L.; CreBie: *Crepis biennis* L.; GalAlb: *Galium album* Mill.; GerPra: *Geranium pratense* L.; HerSph: *Heracleum sphondylium* L.; KnaArv: *Knautia arvensis* (L.) J.M. Coult.; PimMaj: *Pimpinella major* (L.) Huds.; RumAce: *Rumex acetosa* L.; SanOff: *Sanguisorba officinalis* L.; TraPra: *Tragopogon pratensis* L.

Legumes (LE): LatPra: *Lathyrus pratensis* L.; LotCor: *Lotus corniculatus* L.; MedLup: *Medicago lupulina* L.; MedVar: *Medicago x varia* Martyn; OnoVic: *Onobrychis viciifolia* Scop.; TriCam: *Trifolium campestre* Schreb.; TriDub: *Trifolium dubium* Sibth.; TriFra: *Trifolium fragiferum* L.; TriHyb: *Trifolium hybridum* L.; TriPra: *Trifolium pratense* L.; TriRep: *Trifolium repens* L.; VicCra: *Vicia cracca* L.
