# Supplementary material for: Functional composition has stronger impact than species richness on carbon gain and allocation in experimental grasslands
Source: PLoS One. 2019 Jan 31;14(1):e0204715. doi: 10.1371/journal.pone.0204715 (PMC6354960; doi:10.1371/journal.pone.0204715)
Supplement: S2 Table — (PDF) [file pone.0204715.s006.pdf]

S2 Table. Analysis of community-level shoot ^{13}C abundance and ^{13}C excess in relation to functional composition. Shown is the summary of regressions testing for relationships between community-level shoot ^{13}C abundance and ^{13}C excess and functional composition of the mixtures quantified as community-weighted means (CWM) and diversity (FD) of functional traits and canopy leaf nitrogen.

Source of variation	Shoot ^{13}C abundance				Shoot ^{13}C excess			
	Intercept	Predictor	R^2	P	Intercept	Predictor	R^2	P
CWM-N_{Leaf}	33.4385	1.4055	0.502	0.010	8.6210	0.0760	0.017	0.685
CWM-LeafG	46.8498	0.6064	0.098	0.321	2.4290	2.2630	0.013	0.726
CWM-SLA	-11.5100	27.8300	0.127	0.256	5.5740	1.7180	0.006	0.817
CWM-LDMC	66.9181	0.0096	0.001	0.914	10.6043	-0.0002	<0.001	0.993
CWM-g_s	55.6010	0.0275	0.162	0.194	12.6306	-0.0042	0.045	0.509
CWM-H_{Shoot}	73.0256	-0.2287	0.028	0.604	8.7459	0.1057	0.070	0.408
FD-N_{Leaf}	64.0790	10.8970	0.084	0.362	8.0630	5.3790	0.238	0.108
FD-LeafG	77.5810	7.4320	0.278	0.078	12.3140	1.5480	0.141	0.229
FD-SLA	73.8870	3.5790	0.040	0.535	12.5540	1.5040	0.082	0.368
FD-LDMC	66.1670	-2.8130	0.058	0.449	11.5770	0.9770	0.082	0.366
FD-g_s	72.6390	2.4840	0.096	0.326	10.9628	0.2904	0.015	0.701
FD-H_{Shoot}	67.2370	-1.5880	0.008	0.778	13.4920	2.4810	0.237	0.108
Canopy N_{Leaf}	63.5750	2.1900	0.036	0.554	3.1822	2.9104	0.747	<0.001

Abbreviations are: CWM = community-weighted mean, FD = functional diversity, g_s = stomatal conductance, H_{Shoot} = shoot height, LeafG = leaf greenness, LDMC = leaf dry matter content, N_{Leaf} = leaf nitrogen concentration, SLA = specific leaf area
