# Supplementary material for: Functional composition has stronger impact than species richness on carbon gain and allocation in experimental grasslands
Source: PLoS One. 2019 Jan 31;14(1):e0204715. doi: 10.1371/journal.pone.0204715 (PMC6354960; doi:10.1371/journal.pone.0204715)
Supplement: S3 Table — (PDF) [file pone.0204715.s007.pdf]

S3 Table. Analysis of leaf non-structural carbohydrate concentrations and $\delta^{13}C$ in relation to species richness and functional groups. Shown is the summary of mixed-effects models for ^{13}C abundances, ^{13}C atom% excess and concentrations in non-structural carbohydrates (= NSC) in leaves testing for differences between levels of plant species richness, functional groups and different non-structural carbohydrates (glucose, fructose, sucrose, RFO, starch).

Source of variation	^{13}C abundance		^{13}C atom% excess		Concentration	
**Model A**	χ^2	P	χ^2	P	χ^2	P
Species richness (SR)	2.505	0.114	0.768	0.381	0.632	0.427
Functional group identity (FG-ID)	2.732	0.435	8.008	0.046	0.138	0.987
NSC-ID	92.849	<0.001	73.863	<0.001	83.150	<0.001
NSC-ID x SR	1.036	0.904	8.408	0.078	1.247	0.870
NSC-ID x FG-ID	82.611	<0.001	22.556	0.032	95.490	<0.001
**Model B**	χ^2	P	χ^2	P	χ^2	P
Species richness (SR)	2.481	0.115	0.648	0.421	0.531	0.466
Functional group identity (FG-ID)	2.319	0.314	6.109	0.047	0.127	0.939
SR x FG.ID	0.162	0.922	3.238	0.198	0.195	0.907
NSC-ID	82.093	<0.001	64.129	<0.001	75.532	<0.001
NSC-ID x SR	0.855	0.931	10.307	0.036	1.555	0.817
NSC-ID x FG-ID	72.954	<0.001	16.329	0.038	83.210	<0.001
NSC-ID x SR x FG-ID	3.834	0.872	3.317	0.913	3.926	0.864

Models were fitted by stepwise inclusion of fixed effects. Listed are the results of likelihood ratio tests (χ^2) that were applied to assess model improvement and the statistical significance of the fixed effects (P values). Note that the interaction SR × FG-ID and NSC-ID × SR × FG-ID could not be tested in Model A with all samples because we did not sample leaves of tall herbs in the 4-species mixtures. Therefore, the dataset in Model B was reduced and contained only samples of those functional groups, which had been sampled on both diversity levels (grasses, small herbs, and legumes).
