# Supplementary material for: Functional composition has stronger impact than species richness on carbon gain and allocation in experimental grasslands
Source: PLoS One. 2019 Jan 31;14(1):e0204715. doi: 10.1371/journal.pone.0204715 (PMC6354960; doi:10.1371/journal.pone.0204715)
Supplement: S4 Table — (PDF) [file pone.0204715.s008.pdf]

**S4 Table. Analysis of leaf non-structural carbohydrate concentrations and δ$^{13}$C in relation to functional traits.** Shown is the summary of mixed-effects models testing for relationships between $^{13}$C abundance, $^{13}$C atom% excess and concentrations of non-structural carbohydrates (glucose, fructose, sucrose, RFO, starch) in leaves and functional traits.

| Source of variation | Fixed effects | | | | | Random effects | | | AIC |
|---|---|---|---|---|---|---|---|---|---|
| | Intercept | $N_{Leaf}$ | SLA | LDMC | $H_{Shoot}$ | Species ID | Dome | Residuals | |
| **$^{13}$C abundance** | | | | | | | | | |
| Glucose | 1.75570 | | | -0.00399 | | 0.2823 | 0.1771 | 0.3275 | 53.00 |
| Fructose | 0.49931 | 0.72243 | | -0.00343 | | 0.2036 | <0.0001 | 0.4862 | 64.60 |
| Sucrose | -0.09586 | | | 0.00708 | | 0.6364 | 0.1687 | 0.1449 | 57.70 |
| RFO | 5.85530 | | -1.42130 | | | 0.6000 | <0.0001 | 0.3687 | 74.50 |
| Starch | 2.86701 | 0.85639 | | -0.00562 | | 0.4490 | 0.1585 | 0.2237 | 54.80 |
| **$^{13}$C atom% excess** | | | | | | | | | |
| Glucose | 0.03949 | 0.01047 | -0.00843 | | | 0.0025 | 0.0023 | 0.0019 | -286.20 |
| Fructose | 0.04838 | 0.00743 | -0.00980 | | | 0.0025 | 0.0014 | 0.0022 | -279.50 |
| Sucrose | 0.04535 | 0.00687 | -0.00767 | | | 0.0013 | <0.0001 | 0.0019 | -315.40 |
| RFO | 0.07041 | 0.01053 | 0.01536 | 0.00004 | | 0.0027 | <0.0001 | 0.0024 | -286.50 |
| Starch | 0.01904 | 0.00390 | | | | 0.0028 | 0.0015 | 0.0011 | -303.40 |
| **Concentration** | | | | | | | | | |
| Glucose | 3.46528 | | | -0.00349 | -0.02052 | 0.3479 | 0.1405 | 0.2367 | 46.90 |
| Fructose | 2.19079 | 0.50321 | | -0.00401 | | 0.2501 | <0.0001 | 0.4367 | 60.90 |
| Sucrose | 1.25807 | | | 0.00679 | | 0.5260 | 0.1226 | 0.1728 | 50.40 |
| RFO | 2.67388 | -0.96833 | | 0.00493 | | 0.5758 | <0.0001 | 0.3449 | 72.70 |
| Starch | 15.10309 | | | -0.02204 | | 2.1243 | <0.0001 | 0.7163 | 143.70 |

The adequate set of predictor variables was determined by stepwise inclusion of predictor variables and model comparisons. Estimated coefficients are given for the resulting best model. Intercepts and slopes are shown for the fixed effects, and estimated standard deviations are given for the random effects. Abbreviations of functional traits: $N_{Leaf}$ = leaf nitrogen concentration, SLA = specific leaf area, LDMC = leaf dry matter content, $H_{Shoot}$ = shoot height.
