# Supplementary material for: Functional composition has stronger impact than species richness on carbon gain and allocation in experimental grasslands
Source: PLoS One. 2019 Jan 31;14(1):e0204715. doi: 10.1371/journal.pone.0204715 (PMC6354960; doi:10.1371/journal.pone.0204715)
Supplement: S5 Table — (PDF) [file pone.0204715.s009.pdf]

S5 Table. Analysis of functional traits in relation to species richness and functional group identity. Shown is the summary of mixed-effects models testing for effects of species richness (4- and 16-species mixtures) and functional group identity on leaf traits (N_{Leaf} = leaf nitrogen concentration, LeafG = leaf greenness, SLA = specific leaf area, LDMC = leaf dry matter content, g_s = stomatal conductance), and shoot height (H_{Shoot}).

Source of variation		N_{Leaf} (mg g^{-1})			LeafG			g_s (mmol m^{-2} s^{-1})		
	df	χ^2	P		χ^2	P		χ^2	P	
Species richness (SR)	1	2.45	0.118		1.56	0.211		1.47	0.225	
Functional group identity (FG-ID)	3	30.06	<0.001		20.18	<0.001		30.64	<0.001	
SR x FG-ID	3	4.69	0.196		3.47	0.325		4.63	0.201	
**Functional group differences**		mean	sd		mean	sd		mean	sd	
Grasses		19.7	± 6.6	b	30.3	± 6.1	b	202	± 222	b
Small herbs		17.0	± 4.2	b	31.4	± 6.7	b	834	± 368	a
Tall herbs		21.4	± 5.5	b	32.5	± 6.6	b	545	± 170	a
Legumes		34.7	± 8.9	a	44.6	± 9.7	a	421	± 201	a

		SLA (mm^2 mg^{-1})			LDMC (mg g^{-1})			H_{Shoot} (cm)		
	df	χ^2	P		χ^2	P		χ^2	P	
Species richness (SR)	1	1.35	0.244		<0.01	0.969		0.06	0.807	
Functional group identity (FG-ID)	3	6.83	0.077		46.04	<0.001		11.30	0.010	
SR x FG-ID	3	2.29	0.514		4.64	0.200		7.66	0.054	
**Functional group differences**		mean	sd		mean	sd		mean	sd	
Grasses		15.8	± 4.7		312	± 33	a	18	± 10	a
Small herbs		19.9	± 6.6		195	± 39	c	7	± 5	b
Tall herbs		18.8	± 4.8		198	± 48	c	14	± 9	ab
Legumes		20.3	± 6.7		244	± 33	b	18	± 12	ab

Models were fitted by stepwise inclusion of fixed effects. Listed are the results of likelihood ratio tests (χ^2) that were applied to assess model improvement and the statistical significance of the fixed effects (P values). Results of Tukey`s test applied to test for differences among functional groups are indicated with letters.
