# Supplementary material for: Functional composition has stronger impact than species richness on carbon gain and allocation in experimental grasslands
Source: PLoS One. 2019 Jan 31;14(1):e0204715. doi: 10.1371/journal.pone.0204715 (PMC6354960; doi:10.1371/journal.pone.0204715)
Supplement: S6 Table — (PDF) [file pone.0204715.s010.pdf]

S6 Table. Data on species-level shoot biomass and δ^{13}C in shoot biomass. The following variables are listed:
Dome = experimental unit of the Ecotron experiment
Species = species identity (for abbreviations of species names see S1 Table)
FG = species assignment to functional groups (GR = grasses, SH = small herbs, TH = tall herbs, LE = legumes)
SR = sown species richness
BM (g_{dw} m^{-2}) = species-specific shoot biomass based on harvest after the labelling period
δ^{13}C background (‰) = shoot δ^{13}C background values
δ^{13}C uncorr (‰) = measured δ^{13}C values of shoot samples taken after the labelling period
δ^{13}C corr (‰) = δ^{13}C values of labelled shoot samples corrected with a dome-specific factor to account for differences among the experimental units in average air $\delta^{13}C$-CO_2 values during the depletion and enrichment phase
C (mg g_{dw}^{-1}) = shoot carbon concentrations of samples taken after the labelling period based on dry weight
^{13}C atom% excess = ^{13}C atom% excess based on corrected δ^{13}C values
^{13}C abundance (µg g_{dw}^{-1}) = ^{13}C abundance based on corrected δ^{13}C values
^{13}C excess (mg m^{-2}) = ^{13}C excess based on corrected δ^{13}C values
Calculations are described in detail in Material and Methods. Note that for some species with very low abundances δ^{13}C in shoot biomass could not be analysed, while shoot material for δ^{13}C analyses for a few other species could be sampled outside the area used for harvesting shoot biomass (NA = no data available)

Dome	Species	FG	SR	BM (g_{dw} m^{-2})	δ^{13}C background (‰)	δ^{13}C uncorr (‰)	δ^{13}C corr (‰)	C (mg g_{dw}^{-1})	^{13}C atom% excess	^{13}C abundance (µg g_{dw}^{-1})	^{13}C excess (mg m^{-2})
D01	AchMil	TH	16	49.279	-31.635	-12.793	-13.915	420.97	0.019385	81.61	4.021
D01	CenJac	TH	16	NA	-29.285	-15.501	-16.605	418.42	0.013872	58.04	NA
D01	FesPra	GR	16	47.905	-28.302	-14.696	-15.800	423.41	0.013677	57.91	2.774
D01	LatPra	LE	16	29.178	-28.341	-15.467	-16.571	426.20	0.012877	54.88	1.601
D01	LotCor	LE	16	0.319	NA	NA	NA	NA	NA	NA	NA
D01	OnoVic	LE	16	27.533	-28.204	-9.933	-11.037	452.76	0.018779	85.02	2.341
D01	PhlPra	GR	16	NA	-28.330	-17.768	-18.872	439.96	0.010347	45.52	NA
D01	PoaTri	GR	16	0.426	-29.706	-21.337	-22.441	424.19	0.007948	33.71	0.014
D01	RumAce	TH	16	43.653	-28.937	-6.611	-7.715	415.72	0.023215	96.51	4.213
D01	TriFla	GR	16	19.743	-29.332	-13.957	-15.061	430.67	0.015613	67.24	1.328
D01	TriRep	LE	16	11.693	-28.331	-22.648	-23.752	431.31	0.005009	21.61	0.253
D01	VicCra	LE	16	0.972	-28.382	-5.398	-6.503	460.17	0.023934	110.14	0.107
D02	ArrEla	GR	4	157.766	-29.447	-19.625	-19.601	418.72	0.010772	45.10	7.116
D02	PlaLan	SH	4	16.125	-30.136	-14.555	-14.531	432.84	0.017071	73.89	1.192
D02	TriCam	LE	4	0.057	-27.625	NA	NA	NA	NA	NA	NA
D03	AjuRep	SH	16	NA	-28.214	-21.848	-21.763	389.32	0.007058	27.48	NA
D03	AntOdo	GR	16	0.178	-29.392	NA	NA	NA	NA	NA	NA
D03	GerPra	TH	16	91.743	-28.989	-11.271	-11.186	403.14	0.019475	78.51	7.203

S6 Table continued

Dome	Species	FG	SR	BM (g_{dw} m^{-2})	$\delta^{13}C$ background (‰)	$\delta^{13}C$ uncorr (‰)	$\delta^{13}C$ corr (‰)	C (mg g_{dw}^{-1})	^{13}C atom% excess	^{13}C abundance (µg g_{dw}^{-1})	^{13}C excess (mg m^{-2})
D03	HelPub	GR	16	10.271	-29.420	-17.603	-17.519	388.57	0.013020	50.59	0.520
D03	LatPra	LE	16	29.840	-30.362	-15.966	-15.881	420.71	0.015842	66.65	1.989
D03	LotCor	LE	16	1.709	-31.289	NA	NA	NA	NA	NA	NA
D03	PlaLan	SH	16	4.545	-30.962	-18.817	-18.732	387.50	0.013380	51.85	0.236
D03	PoaPra	GR	16	1.374	-31.002	NA	NA	NA	NA	NA	NA
D03	RanRep	SH	16	NA	-29.909	-16.624	-16.539	382.57	0.014627	55.96	NA
D03	TarOff	SH	16	18.364	-30.817	-18.476	-18.391	364.97	0.013593	49.61	0.911
D03	VicCra	LE	16	0.020	-30.887	NA	NA	NA	NA	NA	NA
D04	FesPra	GR	4	10.069	-27.125	-14.963	-14.219	371.39	0.014118	52.43	0.528
D04	PlaLan	SH	4	52.721	-29.309	-10.653	-9.908	364.04	0.021223	77.26	4.073
D05	RanRep	SH	4	138.311	-28.435	-13.280	-14.123	397.36	0.015657	62.21	8.605
D05	TriFra	LE	4	13.630	-29.128	-11.243	-12.085	414.47	0.018644	77.27	1.053
D06	AjuRep	SH	16	5.133	-27.225	-17.648	-18.499	361.65	0.009546	34.52	0.177
D06	AloPra	GR	16	0.891	-28.824	-19.085	-19.936	381.75	0.009724	37.12	0.033
D06	CamPat	TH	16	0.188	-27.507	-15.669	-16.520	358.69	0.012020	43.11	0.008
D06	CynCri	GR	16	0.945	NA	NA	NA	NA	NA	NA	NA
D06	GerPra	TH	16	11.505	-28.285	-11.882	-12.733	430.86	0.017013	73.30	0.843
D06	PlaMed	SH	16	168.063	-29.368	-14.317	-15.168	401.92	0.015535	62.44	10.493
D06	PoaPra	GR	16	0.788	-28.052	-17.116	-17.966	417.70	0.011033	46.08	0.036
D06	RanRep	SH	16	0.123	-28.805	NA	NA	NA	NA	NA	NA
D06	TriDub	LE	16	NA	-27.982	-3.745	-4.596	442.25	0.025581	113.13	NA
D07	AloPra	GR	16	7.610	-28.673	-7.168	-7.083	432.07	0.023617	102.04	0.777
D07	CreBie	TH	16	3.792	-30.315	-18.126	-18.041	400.11	0.013427	53.72	0.204
D07	HerSph	TH	16	0.040	-29.398	-9.012	-8.927	432.14	0.022393	96.77	0.004
D07	LatPra	LE	16	104.194	-28.992	-12.641	-12.556	471.28	0.017980	84.74	8.829
D07	OnoVic	LE	16	4.697	-29.612	-12.302	-12.217	455.05	0.019029	86.59	0.407
D07	PimMaj	TH	16	7.648	-29.011	-9.164	-9.079	422.47	0.021804	92.12	0.705
D07	PlaMed	SH	16	27.760	-29.755	-15.459	-15.374	403.41	0.015733	63.47	1.762
D07	TarOff	SH	16	10.998	-29.735	-16.641	-16.556	415.92	0.014417	59.97	0.659
D07	VerCha	SH	16	1.444	-28.891	-15.226	-15.141	410.04	0.015043	61.68	0.089
D08	AntOdo	GR	4	5.685	-28.628	-13.706	-13.642	390.27	0.016394	63.98	0.364
D08	KnaArv	TH	4	66.772	-28.745	-15.606	-15.542	392.72	0.014444	56.72	3.787
D08	PruVul	SH	4	38.800	-29.899	-14.477	-14.413	388.45	0.016942	65.81	2.553
D08	TriPra	LE	4	0.053	-29.222	NA	NA	NA	NA	NA	NA

S6 Table continued

Dome	Species	FG	SR	BM (g_{dw} m^{-2})	$\delta^{13}C$ background (‰)	$\delta^{13}C$ uncorr (‰)	$\delta^{13}C$ corr (‰)	C (mg g_{dw}^{-1})	^{13}C atom% excess	^{13}C abundance (µg g_{dw}^{-1})	^{13}C excess (mg m^{-2})
D09	AjuRep	SH	16	5.125	-28.256	-20.741	-19.816	380.23	0.009233	35.11	0.180
D09	BelPer	SH	16	1.838	-30.689	-16.676	-15.751	394.33	0.016342	64.44	0.118
D09	BroEre	GR	16	NA	-29.800	-19.068	-18.143	411.57	0.012753	52.49	NA
D09	CreBie	TH	16	55.430	-29.634	-14.836	-13.911	412.45	0.017200	70.94	3.932
D09	FesRub	GR	16	0.782	-27.879	-19.395	-18.470	425.52	0.010293	43.80	0.034
D09	GalAlb	TH	16	10.956	-31.350	-17.498	-16.573	430.46	0.016166	69.59	0.762
D09	GerPra	TH	16	17.204	-27.193	-10.167	-9.242	434.43	0.019636	85.30	1.468
D09	OnoVic	LE	16	NA	-28.241	-11.782	-10.857	443.05	0.019017	84.25	NA
D09	RanRep	SH	16	1.107	-29.507	-13.713	-12.788	417.64	0.018290	76.39	0.085
D09	RumAce	TH	16	1.580	-28.417	-12.694	-11.769	422.34	0.018211	76.91	0.122
D09	VerCha	SH	16	6.384	-29.346	-24.366	-23.442	431.76	0.006459	27.89	0.178
D09	VicCra	LE	16	10.893	-28.431	-12.264	-11.339	453.05	0.018697	84.71	0.923
D10	KnaArv	TH	4	17.576	-28.160	-10.205	-10.223	400.78	0.019621	78.64	1.382
D10	PlaMed	SH	4	166.032	-29.111	-14.289	-14.307	367.27	0.016195	59.48	9.875
D10	VicCra	LE	4	36.311	-28.847	-6.376	-6.394	408.23	0.024560	100.26	3.641
D11	AjuRep	SH	16	0.125	-28.407	-18.953	-18.603	410.02	0.010725	43.98	0.006
D11	ArrEla	GR	16	85.004	-30.044	-15.856	-15.506	429.82	0.015905	68.36	5.811
D11	FesPra	GR	16	NA	-28.796	-18.376	-18.025	416.36	0.011783	49.06	NA
D11	GleHed	SH	16	15.002	-30.504	-21.862	-21.512	408.43	0.009838	40.18	0.603
D11	HelPub	GR	16	44.044	-29.404	-16.814	-16.464	433.92	0.014157	61.43	2.706
D11	LotCor	LE	16	0.947	NA	NA	NA	NA	NA	NA	NA
D11	MedVar	LE	16	NA	-31.208	-12.829	-12.479	439.33	0.020488	90.01	NA
D11	PoaTri	GR	16	0.909	-30.348	-27.445	-27.095	415.19	0.003558	14.77	0.013
D11	RanRep	SH	16	0.691	-30.076	-16.665	-16.315	420.91	0.015054	63.36	0.044
D11	TarOff	SH	16	13.796	-30.214	-17.113	-16.763	412.51	0.014716	60.71	0.837
D11	TriPra	LE	16	NA	-29.887	-7.724	-7.374	435.74	0.024627	107.31	NA
D11	VicCra	LE	16	35.188	-29.872	-12.804	-12.454	441.32	0.019055	84.09	2.959
D12	HerSph	TH	4	2.523	-30.704	-9.356	-8.610	415.58	0.024169	100.44	0.253
D12	MedVar	LE	4	59.438	-30.002	-12.555	-11.809	421.68	0.019902	83.92	4.988
D12	TraPra	TH	4	0.091	NA	NA	NA	NA	NA	NA	NA
D12	TriFla	GR	4	31.206	-29.840	-5.547	-4.801	454.78	0.027388	124.56	3.887
