# Supplementary material for: Functional composition has stronger impact than species richness on carbon gain and allocation in experimental grasslands
Source: PLoS One. 2019 Jan 31;14(1):e0204715. doi: 10.1371/journal.pone.0204715 (PMC6354960; doi:10.1371/journal.pone.0204715)
Supplement: S7 Table — (PDF) [file pone.0204715.s011.pdf]

S7 Table. Data on non-structural carbohydrate concentrations and δ^{13}C in bulk shoot and root samples. The following variables are listed:
Dome = experimental unit of the Ecotron experiment
SR = sown species richness
NSC = non-structural carbohydrate
Comp = plant compartment (shoot, root 0-5 cm and 5-10 cm depth)
δ^{13}C background (‰) = δ^{13}C background values (taken from shoot samples weighted by species biomass proportions)
BM_root (g_{dw} m^{-2}) = root biomass
C (µg C mg_{dw}^{-1}) = carbon concentration based on plant dry weight
C corr (µg C mg_{dw}^{-1}) = corrected carbon concentration according to the C ratio of bulk root biomass using the C ratio of bulk shoot biomass
δ^{13}C uncorr (‰) = measured δ^{13}C of samples taken after the labelling period
δ^{13}C corr (‰) = δ^{13}C of labelled samples corrected with a dome-specific factor to account for differences among the experimental units in average air δ^{13}C-CO_2 values during the depletion and enrichment phase
^{13}C atom% excess = ^{13}C atom% excess based on corrected δ^{13}C values
^{13}C abundance (ng ^{13}C mg_{dw}^{-1}) = ^{13}C abundance based on corrected δ^{13}C values and corrected C concentrations
Calculations are described in detail in Material and Methods. NA = no data available

Dome	SR	Comp	NSC	δ^{13}C background (‰)	BM_root (g_{dw} m^{-2})	C (µg C mg_{dw}^{-1})	C corr (µg C mg_{dw}^{-1})	δ^{13}C uncorr (‰)	δ^{13}C corr (‰)	^{13}C atom% excess	^{13}C abundance (ng ^{13}C mg_{dw}^{-1})
D01	16	Shoot	Glucose	-29.221	--	5.74	5.74	-13.78	-13.87	0.0168	0.96
D02	4	Shoot	Glucose	-29.510	--	9.60	9.60	-20.97	-20.98	0.0093	0.90
D03	16	Shoot	Glucose	-29.588	--	9.75	9.75	-19.27	-19.36	0.0112	1.09
D04	4	Shoot	Glucose	-28.959	--	20.54	20.54	-13.97	-13.28	0.0171	3.52
D05	4	Shoot	Glucose	-29.452	--	26.01	26.01	-12.92	-14.02	0.0169	4.39
D06	16	Shoot	Glucose	-29.232	--	12.87	12.87	-14.50	-15.57	0.0149	1.92
D07	16	Shoot	Glucose	-29.199	--	11.27	11.27	-15.68	-15.94	0.0145	1.63
D08	4	Shoot	Glucose	-29.141	--	13.07	13.07	-6.01	-5.98	0.0253	3.31
D09	16	Shoot	Glucose	-29.215	--	15.06	15.06	-18.74	-17.83	0.0125	1.88
D10	4	Shoot	Glucose	-28.991	--	14.09	14.09	-17.52	-17.53	0.0125	1.77
D11	16	Shoot	Glucose	-29.917	--	18.13	18.13	-19.98	-19.65	0.0112	2.04
D12	4	Shoot	Glucose	-29.966	--	9.64	9.64	-9.70	-9.00	0.0229	2.21
D01	16	Shoot	Fructose	-29.221	--	7.09	7.09	-13.17	-13.26	0.0175	1.24
D02	4	Shoot	Fructose	-29.510	--	4.21	4.21	-15.35	-15.36	0.0155	0.65
D03	16	Shoot	Fructose	-29.588	--	5.26	5.26	-15.31	-15.40	0.0155	0.82
D04	4	Shoot	Fructose	-28.959	--	9.11	9.11	-9.11	-8.42	0.0225	2.05
D05	4	Shoot	Fructose	-29.452	--	8.87	8.87	-8.99	-10.09	0.0212	1.88
D06	16	Shoot	Fructose	-29.232	--	4.42	4.42	-13.01	-14.08	0.0166	0.73
D07	16	Shoot	Fructose	-29.199	--	3.37	3.37	-6.49	-6.74	0.0246	0.83
D08	4	Shoot	Fructose	-29.141	--	5.22	5.22	-6.20	-6.16	0.0251	1.31

S7 Table continued

Dome	SR	Comp	NSC	δ13C background (‰)	BM_root (g_{dw} m^{-2})	C (µg C mg_{dw}^{-1})	C corr (µg C mg_{dw}^{-1})	δ13C uncorr (‰)	δ13C corr (‰)	13C atom% excess	13C abundance (ng 13C mg_{dw}^{-1})
D09	16	Shoot	Fructose	-29.215	--	11.22	11.22	-13.50	-12.58	0.0182	2.04
D10	4	Shoot	Fructose	-28.991	--	3.72	3.72	-13.11	-13.12	0.0174	0.65
D11	16	Shoot	Fructose	-29.917	--	4.93	4.93	-11.11	-10.78	0.0209	1.03
D12	4	Shoot	Fructose	-29.966	--	6.37	6.37	-8.02	-7.32	0.0248	1.58
D01	16	Shoot	Sucrose	-29.221	--	15.68	15.68	-7.64	-7.73	0.0235	3.69
D02	4	Shoot	Sucrose	-29.510	--	15.61	15.61	-7.71	-7.72	0.0238	3.72
D03	16	Shoot	Sucrose	-29.588	--	17.60	17.60	-12.34	-12.43	0.0188	3.30
D04	4	Shoot	Sucrose	-28.959	--	10.84	10.84	-7.90	-7.22	0.0238	2.58
D05	4	Shoot	Sucrose	-29.452	--	14.64	14.64	-3.84	-4.94	0.0268	3.93
D06	16	Shoot	Sucrose	-29.232	--	9.06	9.06	-7.66	-8.73	0.0224	2.03
D07	16	Shoot	Sucrose	-29.199	--	7.53	7.53	-5.82	-6.08	0.0253	1.91
D08	4	Shoot	Sucrose	-29.141	--	13.62	13.62	-4.69	-4.65	0.0268	3.65
D09	16	Shoot	Sucrose	-29.215	--	19.44	19.44	-5.67	-4.76	0.0268	5.20
D10	4	Shoot	Sucrose	-28.991	--	4.85	4.85	-9.04	-9.05	0.0218	1.06
D11	16	Shoot	Sucrose	-29.917	--	14.30	14.30	-4.85	-4.53	0.0278	3.97
D12	4	Shoot	Sucrose	-29.966	--	22.97	22.97	-4.50	-3.80	0.0286	6.58
D01	16	Shoot	RFO	-29.221	--	39.42	39.42	-12.23	-12.32	0.0185	7.29
D02	4	Shoot	RFO	-29.510	--	27.33	27.33	-15.29	-15.30	0.0155	4.25
D03	16	Shoot	RFO	-29.588	--	25.91	25.91	-23.19	-23.29	0.0069	1.79
D04	4	Shoot	RFO	-28.959	--	8.99	8.99	-10.58	-9.90	0.0209	1.87
D05	4	Shoot	RFO	-29.452	--	13.87	13.87	-9.90	-11.00	0.0202	2.80
D06	16	Shoot	RFO	-29.232	--	18.63	18.63	-7.78	-8.86	0.0223	4.15
D07	16	Shoot	RFO	-29.199	--	6.26	6.26	-10.49	-10.75	0.0202	1.26
D08	4	Shoot	RFO	-29.141	--	45.68	45.68	-6.28	-6.24	0.0250	11.44
D09	16	Shoot	RFO	-29.215	--	29.02	29.02	-10.82	-9.90	0.0211	6.13
D10	4	Shoot	RFO	-28.991	--	6.33	6.33	-14.40	-14.40	0.0160	1.01
D11	16	Shoot	RFO	-29.917	--	28.92	28.92	-12.50	-12.17	0.0194	5.61
D12	4	Shoot	RFO	-29.966	--	26.79	26.79	-5.24	-4.54	0.0278	7.45
D01	16	Shoot	Starch	-29.221	--	6.67	6.67	-12.77	-12.86	0.0179	1.19
D02	4	Shoot	Starch	-29.510	--	7.82	7.82	-13.84	-13.85	0.0171	1.34
D03	16	Shoot	Starch	-29.588	--	6.82	6.82	-18.54	-18.63	0.0120	0.82
D04	4	Shoot	Starch	-28.959	--	8.60	8.60	-13.73	-13.04	0.0174	1.50
D05	4	Shoot	Starch	-29.452	--	10.25	10.25	-14.01	-15.11	0.0157	1.61
D06	16	Shoot	Starch	-29.232	--	8.67	8.67	-12.95	-14.03	0.0166	1.44

S7 Table continued

Dome	SR	Comp	NSC	δ^{13}C background (‰)	BM_root (g_{dw} m^{-2})	C (µg C mg_{dw}^{-1})	C corr (µg C mg_{dw}^{-1})	δ^{13}C uncorr (‰)	δ^{13}C corr (‰)	^{13}C atom% excess	^{13}C abundance (ng ^{13}C mg_{dw}^{-1})
D07	16	Shoot	Starch	-29.199	--	9.29	9.29	-11.09	-11.35	0.0195	1.81
D08	4	Shoot	Starch	-29.141	--	10.79	10.79	-11.82	-11.79	0.0190	2.05
D09	16	Shoot	Starch	-29.215	--	10.36	10.36	-13.36	-12.45	0.0183	1.90
D10	4	Shoot	Starch	-28.991	--	6.84	6.84	-11.73	-11.74	0.0189	1.29
D11	16	Shoot	Starch	-29.917	--	5.50	5.50	-14.31	-13.98	0.0174	0.96
D12	4	Shoot	Starch	-29.966	--	9.10	9.10	-9.68	-8.98	0.0230	2.09
D01	16	Root_0-5	Glucose	-29.221	398.80	1.23	1.31	-18.85	-18.94	0.0112	0.15
D02	4	Root_0-5	Glucose	-29.510	165.10	NA	NA	NA	NA	NA	NA
D03	16	Root_0-5	Glucose	-29.588	294.25	1.43	3.02	-28.64	-28.73	0.0009	0.03
D04	4	Root_0-5	Glucose	-28.959	94.45	4.62	6.62	-24.33	-23.64	0.0058	0.39
D05	4	Root_0-5	Glucose	-29.452	575.50	14.64	21.89	-17.89	-19.00	0.0114	2.50
D06	16	Root_0-5	Glucose	-29.232	370.25	1.84	2.49	-22.66	-23.73	0.0060	0.15
D07	16	Root_0-5	Glucose	-29.199	582.90	1.09	1.49	-19.35	-19.60	0.0105	0.16
D08	4	Root_0-5	Glucose	-29.141	121.65	0.93	1.82	-15.63	-15.60	0.0148	0.27
D09	16	Root_0-5	Glucose	-29.215	372.25	NA	NA	NA	NA	NA	NA
D10	4	Root_0-5	Glucose	-28.991	259.45	5.71	8.02	-23.50	-23.50	0.0060	0.48
D11	16	Root_0-5	Glucose	-29.917	694.45	NA	NA	NA	NA	NA	NA
D12	4	Root_0-5	Glucose	-29.966	239.50	3.46	4.73	-24.16	-23.46	0.0071	0.34
D01	16	Root_0-5	Fructose	-29.221	398.80	0.78	0.83	-21.23	-21.32	0.0086	0.07
D02	4	Root_0-5	Fructose	-29.510	165.10	NA	NA	NA	NA	NA	NA
D03	16	Root_0-5	Fructose	-29.588	294.25	NA	NA	NA	NA	NA	NA
D04	4	Root_0-5	Fructose	-28.959	94.45	0.80	1.15	-22.06	-21.38	0.0083	0.10
D05	4	Root_0-5	Fructose	-29.452	575.50	2.20	3.29	-17.33	-18.43	0.0121	0.40
D06	16	Root_0-5	Fructose	-29.232	370.25	0.99	1.34	-17.91	-18.98	0.0112	0.15
D07	16	Root_0-5	Fructose	-29.199	582.90	0.66	0.90	-19.35	-19.61	0.0105	0.09
D08	4	Root_0-5	Fructose	-29.141	121.65	0.79	1.53	-18.65	-18.61	0.0115	0.18
D09	16	Root_0-5	Fructose	-29.215	372.25	NA	NA	NA	NA	NA	NA
D10	4	Root_0-5	Fructose	-28.991	259.45	2.02	2.83	-13.91	-13.92	0.0165	0.47
D11	16	Root_0-5	Fructose	-29.917	694.45	NA	NA	NA	NA	NA	NA
D12	4	Root_0-5	Fructose	-29.966	239.50	2.09	2.86	-22.61	-21.91	0.0088	0.25
D01	16	Root_0-5	Sucrose	-29.221	398.80	6.31	6.75	-12.14	-12.22	0.0186	1.26
D02	4	Root_0-5	Sucrose	-29.510	165.10	5.29	7.47	-10.54	-10.55	0.0207	1.55
D03	16	Root_0-5	Sucrose	-29.588	294.25	3.04	6.41	-22.23	-22.32	0.0080	0.51
D04	4	Root_0-5	Sucrose	-28.959	94.45	4.61	6.61	-14.00	-13.31	0.0171	1.13

S7 Table continued

Dome	SR	Comp	NSC	$\delta^{13}C$ background (‰)	BM_root ($g_{dw}\ m^{-2}$)	C (µg C mg_{dw}^{-1})	C corr (µg C mg_{dw}^{-1})	$\delta^{13}C$ uncorr (‰)	$\delta^{13}C$ corr (‰)	^{13}C atom% excess	^{13}C abundance (ng ^{13}C mg_{dw}^{-1})
D05	4	Root_0-5	Sucrose	-29.452	575.50	4.86	7.27	-7.57	-8.67	0.0227	1.65
D06	16	Root_0-5	Sucrose	-29.232	370.25	3.80	5.14	-12.00	-13.07	0.0177	0.91
D07	16	Root_0-5	Sucrose	-29.199	582.90	7.31	10.00	-11.04	-11.29	0.0196	1.96
D08	4	Root_0-5	Sucrose	-29.141	121.65	1.80	3.52	-12.20	-12.17	0.0186	0.65
D09	16	Root_0-5	Sucrose	-29.215	372.25	3.71	5.63	-15.16	-14.24	0.0164	0.92
D10	4	Root_0-5	Sucrose	-28.991	259.45	11.61	16.31	-11.29	-11.30	0.0194	3.16
D11	16	Root_0-5	Sucrose	-29.917	694.45	4.90	6.31	-10.79	-10.47	0.0213	1.34
D12	4	Root_0-5	Sucrose	-29.966	239.50	6.58	8.98	-14.02	-13.32	0.0182	1.64
D01	16	Root_0-5	RFO	-29.221	398.80	32.46	34.74	-22.89	-22.98	0.0068	2.37
D02	4	Root_0-5	RFO	-29.510	165.10	48.92	69.11	-21.47	-21.48	0.0088	6.07
D03	16	Root_0-5	RFO	-29.588	294.25	15.37	32.44	-25.30	-25.39	0.0046	1.49
D04	4	Root_0-5	RFO	-28.959	94.45	25.22	36.13	-17.45	-16.77	0.0133	4.82
D05	4	Root_0-5	RFO	-29.452	575.50	10.96	16.40	-20.51	-21.61	0.0086	1.41
D06	16	Root_0-5	RFO	-29.232	370.25	24.70	33.46	-21.33	-22.40	0.0075	2.50
D07	16	Root_0-5	RFO	-29.199	582.90	10.78	14.75	-23.43	-23.69	0.0060	0.89
D08	4	Root_0-5	RFO	-29.141	121.65	6.65	12.96	-17.96	-17.93	0.0123	1.59
D09	16	Root_0-5	RFO	-29.215	372.25	60.60	92.11	-24.44	-23.53	0.0062	5.73
D10	4	Root_0-5	RFO	-28.991	259.45	15.31	21.50	-21.89	-21.89	0.0078	1.67
D11	16	Root_0-5	RFO	-29.917	694.45	45.51	58.56	-20.56	-20.24	0.0106	6.20
D12	4	Root_0-5	RFO	-29.966	239.50	24.38	33.31	-20.36	-19.66	0.0113	3.76
D01	16	Root_0-5	Starch	-29.221	398.80	5.66	6.06	-25.18	-25.26	0.0043	0.26
D02	4	Root_0-5	Starch	-29.510	165.10	1.39	1.96	-28.00	-28.02	0.0016	0.03
D03	16	Root_0-5	Starch	-29.588	294.25	4.78	10.09	-34.38	-34.47	-0.0053	-0.54
D04	4	Root_0-5	Starch	-28.959	94.45	4.12	5.91	-29.87	-29.19	-0.0002	-0.01
D05	4	Root_0-5	Starch	-29.452	575.50	5.75	8.60	-29.09	-30.19	-0.0008	-0.07
D06	16	Root_0-5	Starch	-29.232	370.25	2.21	2.99	-29.13	-30.21	-0.0011	-0.03
D07	16	Root_0-5	Starch	-29.199	582.90	4.69	6.41	-27.56	-27.82	0.0015	0.10
D08	4	Root_0-5	Starch	-29.141	121.65	2.19	4.27	-26.51	-26.48	0.0029	0.12
D09	16	Root_0-5	Starch	-29.215	372.25	3.49	5.30	-27.28	-26.37	0.0031	0.17
D10	4	Root_0-5	Starch	-28.991	259.45	5.81	8.15	-26.82	-26.82	0.0024	0.19
D11	16	Root_0-5	Starch	-29.917	694.45	3.28	4.22	-26.57	-26.24	0.0040	0.17
D12	4	Root_0-5	Starch	-29.966	239.50	4.28	5.85	-28.66	-27.96	0.0022	0.13
D01	16	Root_5-10	Glucose	-29.221	114.90	0.85	1.27	-26.25	-26.34	0.0032	0.04
D02	4	Root_5-10	Glucose	-29.510	118.85	1.04	2.24	-23.56	-23.57	0.0065	0.15

S7 Table continued

Dome	SR	Comp	NSC	$\delta^{13}C$ background (‰)	BM_root (g_{dw} m^{-2})	C (µg C mg_{dw}^{-1})	C corr (µg C mg_{dw}^{-1})	$\delta^{13}C$ uncorr (‰)	$\delta^{13}C$ corr (‰)	^{13}C atom% excess	^{13}C abundance (ng ^{13}C mg_{dw}^{-1})
D03	16	Root_5-10	Glucose	-29.588	120.35	2.04	5.44	-27.17	-27.26	0.0026	0.14
D04	4	Root_5-10	Glucose	-28.959	95.50	3.32	6.88	-24.27	-23.58	0.0059	0.40
D05	4	Root_5-10	Glucose	-29.452	96.20	9.38	20.78	-12.29	-13.40	0.0176	3.65
D06	16	Root_5-10	Glucose	-29.232	73.10	4.98	6.76	-24.46	-25.53	0.0040	0.27
D07	16	Root_5-10	Glucose	-29.199	229.65	4.80	8.10	-27.40	-27.66	0.0017	0.14
D08	4	Root_5-10	Glucose	-29.141	89.95	0.55	1.13	-16.45	-16.41	0.0139	0.16
D09	16	Root_5-10	Glucose	-29.215	199.70	2.47	4.32	-23.05	-22.14	0.0077	0.33
D10	4	Root_5-10	Glucose	-28.991	148.30	9.15	14.44	-26.10	-26.11	0.0032	0.46
D11	16	Root_5-10	Glucose	-29.917	79.70	0.79	1.18	-18.04	-17.71	0.0134	0.16
D12	4	Root_5-10	Glucose	-29.966	116.90	1.88	2.74	-20.20	-19.50	0.0115	0.31
D01	16	Root_5-10	Fructose	-29.221	114.90	NA	NA	NA	NA	NA	NA
D02	4	Root_5-10	Fructose	-29.510	118.85	NA	NA	NA	NA	NA	NA
D03	16	Root_5-10	Fructose	-29.588	120.35	1.01	2.69	-23.66	-23.75	0.0064	0.17
D04	4	Root_5-10	Fructose	-28.959	95.50	NA	NA	NA	NA	NA	NA
D05	4	Root_5-10	Fructose	-29.452	96.20	1.69	3.73	-18.87	-19.97	0.0104	0.39
D06	16	Root_5-10	Fructose	-29.232	73.10	0.81	1.10	-23.02	-24.09	0.0056	0.06
D07	16	Root_5-10	Fructose	-29.199	229.65	0.49	0.83	-23.56	-23.81	0.0059	0.05
D08	4	Root_5-10	Fructose	-29.141	89.95	NA	NA	NA	NA	NA	NA
D09	16	Root_5-10	Fructose	-29.215	199.70	2.97	5.19	-27.48	-26.57	0.0029	0.15
D10	4	Root_5-10	Fructose	-28.991	148.30	2.17	3.43	-15.47	-15.47	0.0148	0.51
D11	16	Root_5-10	Fructose	-29.917	79.70	1.00	1.48	-18.22	-17.89	0.0132	0.20
D12	4	Root_5-10	Fructose	-29.966	116.90	1.27	1.85	-17.99	-17.29	0.0139	0.26
D01	16	Root_5-10	Sucrose	-29.221	114.90	4.10	6.13	-13.26	-13.35	0.0174	1.06
D02	4	Root_5-10	Sucrose	-29.510	118.85	2.54	5.46	-11.62	-11.63	0.0196	1.07
D03	16	Root_5-10	Sucrose	-29.588	120.35	2.64	7.06	-11.05	-11.14	0.0202	1.42
D04	4	Root_5-10	Sucrose	-28.959	95.50	2.29	4.74	-16.50	-15.81	0.0144	0.68
D05	4	Root_5-10	Sucrose	-29.452	96.20	2.31	5.12	-15.17	-16.27	0.0144	0.74
D06	16	Root_5-10	Sucrose	-29.232	73.10	7.23	9.82	-24.07	-25.14	0.0045	0.44
D07	16	Root_5-10	Sucrose	-29.199	229.65	2.81	4.74	-13.80	-14.06	0.0166	0.79
D08	4	Root_5-10	Sucrose	-29.141	89.95	0.78	1.60	-14.64	-14.60	0.0159	0.25
D09	16	Root_5-10	Sucrose	-29.215	199.70	6.51	11.38	-20.92	-20.01	0.0101	1.15
D10	4	Root_5-10	Sucrose	-28.991	148.30	4.12	6.50	-19.30	-19.30	0.0106	0.69
D11	16	Root_5-10	Sucrose	-29.917	79.70	3.64	5.41	-9.99	-9.66	0.0222	1.20
D12	4	Root_5-10	Sucrose	-29.966	116.90	13.65	19.89	-5.53	-4.83	0.0275	5.47

S7 Table continued

Dome	SR	Comp	NSC	$\delta^{13}C$ background (‰)	BM_root (g_{dw} m^{-2})	C (µg C mg_{dw}^{-1})	C corr (µg C mg_{dw}^{-1})	$\delta^{13}C$ uncorr (‰)	$\delta^{13}C$ corr (‰)	^{13}C atom% excess	^{13}C abundance (ng ^{13}C mg_{dw}^{-1})
D01	16	Root_5-10	RFO	-29.221	114.90	9.81	14.65	-13.50	-13.59	0.0171	2.50
D02	4	Root_5-10	RFO	-29.510	118.85	35.94	77.36	-16.09	-16.10	0.0147	11.35
D03	16	Root_5-10	RFO	-29.588	120.35	17.46	46.64	-22.87	-22.96	0.0073	3.38
D04	4	Root_5-10	RFO	-28.959	95.50	23.25	48.18	-16.74	-16.06	0.0141	6.80
D05	4	Root_5-10	RFO	-29.452	96.20	20.16	44.67	-24.88	-25.98	0.0038	1.70
D06	16	Root_5-10	RFO	-29.232	73.10	30.53	41.47	-17.92	-18.99	0.0112	4.65
D07	16	Root_5-10	RFO	-29.199	229.65	10.45	17.65	-13.85	-14.11	0.0165	2.91
D08	4	Root_5-10	RFO	-29.141	89.95	1.79	3.69	-15.81	-15.78	0.0146	0.54
D09	16	Root_5-10	RFO	-29.215	199.70	58.07	101.55	-27.63	-26.71	0.0027	2.78
D10	4	Root_5-10	RFO	-28.991	148.30	26.74	42.19	-23.95	-23.96	0.0055	2.32
D11	16	Root_5-10	RFO	-29.917	79.70	30.23	44.94	-14.74	-14.42	0.0170	7.62
D12	4	Root_5-10	RFO	-29.966	116.90	12.52	18.24	-15.07	-14.37	0.0171	3.11
D01	16	Root_5-10	Starch	-29.221	114.90	2.94	4.39	-20.85	-20.94	0.0091	0.40
D02	4	Root_5-10	Starch	-29.510	118.85	1.04	2.25	-26.59	-26.60	0.0032	0.07
D03	16	Root_5-10	Starch	-29.588	120.35	5.71	15.26	-33.93	-34.02	-0.0048	-0.74
D04	4	Root_5-10	Starch	-28.959	95.50	2.30	4.77	-29.28	-28.59	0.0004	0.02
D05	4	Root_5-10	Starch	-29.452	96.20	5.85	12.97	-20.81	-21.92	0.0082	1.07
D06	16	Root_5-10	Starch	-29.232	73.10	3.08	4.18	-28.41	-29.49	-0.0003	-0.01
D07	16	Root_5-10	Starch	-29.199	229.65	5.24	8.85	-23.95	-24.21	0.0055	0.48
D08	4	Root_5-10	Starch	-29.141	89.95	2.08	4.30	-27.99	-27.96	0.0013	0.06
D09	16	Root_5-10	Starch	-29.215	199.70	2.80	4.89	-27.61	-26.70	0.0028	0.13
D10	4	Root_5-10	Starch	-28.991	148.30	5.24	8.27	-31.17	-31.18	-0.0024	-0.20
D11	16	Root_5-10	Starch	-29.917	79.70	3.21	4.77	-26.79	-26.47	0.0038	0.18
D12	4	Root_5-10	Starch	-29.966	116.90	6.12	8.92	-21.48	-20.79	0.0100	0.90
