# Supplementary material for: Functional composition has stronger impact than species richness on carbon gain and allocation in experimental grasslands
Source: PLoS One. 2019 Jan 31;14(1):e0204715. doi: 10.1371/journal.pone.0204715 (PMC6354960; doi:10.1371/journal.pone.0204715)
Supplement: S8 Table — (PDF) [file pone.0204715.s012.pdf]

S8 Table. Data on non-structural carbohydrate concentrations and δ^{13}C in leaf samples. The following variables are listed:
Dome = experimental unit of the Ecotron experiment
Species = species identity (for abbreviations of species names see S1 Table)
FG = species assignment to functional groups (GR = grasses, SH = small herbs, TH = tall herbs, LE = legumes)
SR = sown species richness
NSC = non-structural carbohydrate
δ^{13}C background (‰) = δ^{13}C background values (taken from shoot samples)
C (µg C mg_{dw}^{-1}) = carbon concentration based on plant dry weight
δ^{13}C uncorr (‰) = measured δ^{13}C of samples taken after the labelling period
δ^{13}C corr (‰) = δ^{13}C of labelled samples corrected with a dome-specific factor to account for differences among the experimental units in average air δ^{13}C-CO_2 values during the depletion and enrichment phase
^{13}C atom% excess = ^{13}C atom% excess based on corrected δ^{13}C values
^{13}C abundance (ng ^{13}C mg_{dw}^{-1}) = ^{13}C abundance based on corrected δ^{13}C values
Calculations are described in detail in Material and Methods. NA = no data available

Dome	Species	SR	FG	NSC	δ^{13}C background (‰)	C (µg C mg_{dw}^{-1})	δ^{13}C uncorr (‰)	δ^{13}C corr (‰)	^{13}C atom% excess	^{13}C abundance (ng ^{13}C mg_{dw}^{-1})
D01	AchMil	16	TH	Glucose	-31.635	6.48	-2.276	-3.398	0.0309	2.00
D01	AchMil	16	TH	Fructose	-31.635	8.22	-0.919	-2.041	0.0324	2.66
D01	AchMil	16	TH	Sucrose	-31.635	8.30	-1.113	-2.234	0.0322	2.67
D01	AchMil	16	TH	RFO	-31.635	20.48	-3.397	-4.518	0.0297	6.07
D01	AchMil	16	TH	Starch	-31.635	10.49	-7.088	-8.210	0.0256	2.69
D01	FesPra	16	GR	Glucose	-28.302	5.46	0.410	-0.712	0.0302	1.65
D01	FesPra	16	GR	Fructose	-28.302	4.52	2.601	1.479	0.0326	1.47
D01	FesPra	16	GR	Sucrose	-28.302	18.62	2.398	1.276	0.0324	6.03
D01	FesPra	16	GR	RFO	-28.302	81.72	-3.320	-4.442	0.0261	21.33
D01	FesPra	16	GR	Starch	-28.302	9.87	-1.928	-3.050	0.0276	2.73
D01	LatPra	16	LE	Glucose	-28.341	7.78	0.576	-0.546	0.0304	2.37
D01	LatPra	16	LE	Fructose	-28.341	5.37	3.932	2.810	0.0341	1.83
D01	LatPra	16	LE	Sucrose	-28.341	11.29	1.375	0.253	0.0313	3.53
D01	LatPra	16	LE	RFO	-28.341	15.12	-4.198	-5.320	0.0252	3.81
D01	LatPra	16	LE	Starch	-28.341	11.36	-3.982	-5.104	0.0254	2.89
D01	TriFla	16	GR	Glucose	-29.332	4.24	-4.476	-5.598	0.0260	1.10
D01	TriFla	16	GR	Fructose	-29.332	3.35	-0.382	-1.504	0.0304	1.02
D01	TriFla	16	GR	Sucrose	-29.332	42.87	0.134	-0.988	0.0310	13.29
D01	TriFla	16	GR	RFO	-29.332	7.41	3.249	2.127	0.0344	2.55
D01	TriFla	16	GR	Starch	-29.332	9.61	-2.549	-3.671	0.0281	2.70
D02	ArrEla	4	GR	Glucose	-29.447	5.66	-10.375	-10.369	0.0209	1.18

S8 Table continued

Dome	Species	SR	FG	NSC	$\delta^{13}C$ background (‰)	C (µg C mg_{dw}^{-1})	$\delta^{13}C$ uncorr (‰)	$\delta^{13}C$ corr (‰)	^{13}C atom% excess	^{13}C abundance (ng ^{13}C mg_{dw}^{-1})
D02	ArrEla	4	GR	Fructose	-29.447	3.36	-3.911	-3.905	0.0279	0.94
D02	ArrEla	4	GR	Sucrose	-29.447	49.49	-3.688	-3.682	0.0282	13.95
D02	ArrEla	4	GR	RFO	-29.447	16.07	-6.089	-6.083	0.0256	4.11
D02	ArrEla	4	GR	Starch	-29.447	6.66	-7.639	-7.633	0.0239	1.59
D02	PlaLan	4	SH	Glucose	-30.136	20.94	-16.533	-16.527	0.0149	3.12
D02	PlaLan	4	SH	Fructose	-30.136	4.09	-8.582	-8.576	0.0236	0.97
D02	PlaLan	4	SH	Sucrose	-30.136	7.32	-6.894	-6.888	0.0254	1.86
D02	PlaLan	4	SH	RFO	-30.136	21.68	-5.840	-5.834	0.0266	5.76
D02	PlaLan	4	SH	Starch	-30.136	10.99	-11.156	-11.150	0.0208	2.28
D03	GerPra	16	TH	Glucose	-28.989	6.83	-5.698	-5.630	0.0256	1.74
D03	GerPra	16	TH	Fructose	-28.989	4.07	-5.079	-5.011	0.0262	1.07
D03	GerPra	16	TH	Sucrose	-28.989	13.68	-1.891	-1.824	0.0297	4.07
D03	GerPra	16	TH	RFO	-28.989	9.77	-9.234	-9.166	0.0217	2.12
D03	GerPra	16	TH	Starch	-28.989	11.13	-8.193	-8.126	0.0228	2.54
D03	HelPub	16	GR	Glucose	-29.420	6.78	-12.548	-12.480	0.0185	1.26
D03	HelPub	16	GR	Fructose	-29.420	4.81	-6.039	-5.971	0.0256	1.23
D03	HelPub	16	GR	Sucrose	-29.420	27.93	-4.103	-4.036	0.0278	7.75
D03	HelPub	16	GR	RFO	-29.420	41.91	-7.927	-7.860	0.0236	9.88
D03	HelPub	16	GR	Starch	-29.420	3.60	-7.478	-7.411	0.0241	0.87
D03	PlaLan	16	SH	Glucose	-30.962	15.59	-13.802	-13.735	0.0188	2.94
D03	PlaLan	16	SH	Fructose	-30.962	6.60	-7.235	-7.168	0.0260	1.72
D03	PlaLan	16	SH	Sucrose	-30.962	11.44	-7.927	-7.860	0.0253	2.89
D03	PlaLan	16	SH	RFO	-30.962	29.75	-7.731	-7.664	0.0255	7.58
D03	PlaLan	16	SH	Starch	-30.962	10.67	-15.458	-15.391	0.0170	1.82
D03	TarOff	16	SH	Glucose	-30.817	6.69	-16.271	-16.203	0.0160	1.07
D03	TarOff	16	SH	Fructose	-30.817	9.75	-20.171	-20.103	0.0117	1.14
D03	TarOff	16	SH	Sucrose	-30.817	20.64	-4.706	-4.639	0.0286	5.91
D03	TarOff	16	SH	RFO	-30.817	16.19	-19.349	-19.282	0.0126	2.04
D03	TarOff	16	SH	Starch	-30.817	8.61	-13.749	-13.682	0.0187	1.61
D04	FesPra	4	GR	Glucose	-27.125	4.81	-3.640	-2.913	0.0265	1.27
D04	FesPra	4	GR	Fructose	-27.125	4.27	-2.821	-2.094	0.0274	1.17
D04	FesPra	4	GR	Sucrose	-27.125	24.94	-0.586	0.141	0.0298	7.44
D04	FesPra	4	GR	RFO	-27.125	124.51	-2.297	-1.570	0.0280	34.80
D04	FesPra	4	GR	Starch	-27.125	7.52	-4.068	-3.341	0.0260	1.96

S8 Table continued

Dome	Species	SR	FG	NSC	$\delta^{13}C$ background (‰)	C (µg C mg_{dw}^{-1})	$\delta^{13}C$ uncorr (‰)	$\delta^{13}C$ corr (‰)	^{13}C atom% excess	^{13}C abundance (ng ^{13}C mg_{dw}^{-1})
D04	PlaLan	4	SH	Glucose	-29.309	25.41	-8.651	-7.924	0.0234	5.94
D04	PlaLan	4	SH	Fructose	-29.309	6.57	-2.651	-1.924	0.0300	1.97
D04	PlaLan	4	SH	Sucrose	-29.309	11.78	-1.311	-0.584	0.0314	3.70
D04	PlaLan	4	SH	RFO	-29.309	17.09	-0.924	-0.197	0.0318	5.44
D04	PlaLan	4	SH	Starch	-29.309	10.69	-8.116	-7.389	0.0240	2.56
D05	RanRep	4	SH	Glucose	-28.435	26.48	0.356	-0.505	0.0305	8.09
D05	RanRep	4	SH	Fructose	-28.435	8.80	-2.472	-3.332	0.0275	2.42
D05	RanRep	4	SH	Sucrose	-28.435	26.73	0.583	-0.278	0.0308	8.23
D05	RanRep	4	SH	RFO	-28.435	13.03	2.747	1.887	0.0332	4.32
D05	RanRep	4	SH	Starch	-28.435	11.03	-5.732	-6.592	0.0239	2.63
D06	GerPra	16	TH	Glucose	-28.285	7.97	-4.596	-5.464	0.0250	1.99
D06	GerPra	16	TH	Fructose	-28.285	3.77	-8.192	-9.060	0.0210	0.79
D06	GerPra	16	TH	Sucrose	-28.285	23.95	-1.217	-2.086	0.0287	6.86
D06	GerPra	16	TH	RFO	-28.285	6.59	-3.616	-4.485	0.0260	1.72
D06	GerPra	16	TH	Starch	-28.285	10.27	-7.246	-8.115	0.0221	2.27
D06	PlaMed	16	SH	Glucose	-29.368	12.50	-0.366	-1.234	0.0308	3.85
D06	PlaMed	16	SH	Fructose	-29.368	6.45	1.208	0.339	0.0325	2.10
D06	PlaMed	16	SH	Sucrose	-29.368	4.05	-0.405	-1.273	0.0307	1.25
D06	PlaMed	16	SH	RFO	-29.368	8.84	0.125	-0.744	0.0313	2.77
D06	PlaMed	16	SH	Starch	-29.368	11.20	-4.945	-5.814	0.0258	2.89
D07	AloPra	16	GR	Glucose	-28.673	3.44	-12.112	-12.045	0.0182	0.63
D07	AloPra	16	GR	Fructose	-28.673	2.35	-10.852	-10.784	0.0196	0.46
D07	AloPra	16	GR	Sucrose	-28.673	25.31	-2.657	-2.589	0.0285	7.22
D07	AloPra	16	GR	RFO	-28.673	71.16	-14.727	-14.659	0.0153	10.91
D07	AloPra	16	GR	Starch	-28.673	3.17	-12.835	-12.768	0.0174	0.55
D07	LatPra	16	LE	Glucose	-28.992	10.41	-3.795	-3.727	0.0276	2.88
D07	LatPra	16	LE	Fructose	-28.992	10.72	-5.087	-5.019	0.0262	2.81
D07	LatPra	16	LE	Sucrose	-28.992	13.85	-1.749	-1.681	0.0299	4.14
D07	LatPra	16	LE	RFO	-28.992	11.37	-3.918	-3.850	0.0275	3.13
D07	LatPra	16	LE	Starch	-28.992	11.48	-10.103	-10.036	0.0207	2.38
D07	PimMaj	16	TH	Glucose	-29.011	7.84	-10.833	-10.766	0.0200	1.57
D07	PimMaj	16	TH	Fructose	-29.011	5.42	-7.232	-7.165	0.0239	1.29
D07	PimMaj	16	TH	Sucrose	-29.011	17.60	-3.450	-3.382	0.0280	4.93
D07	PimMaj	16	TH	RFO	-29.011	21.87	-3.792	-3.725	0.0277	6.05

S8 Table continued

Dome	Species	SR	FG	NSC	δ^{13}C background (‰)	C (µg C mg_{dw}^{-1})	δ^{13}C uncorr (‰)	δ^{13}C corr (‰)	^{13}C atom% excess	^{13}C abundance (ng ^{13}C mg_{dw}^{-1})
D07	PimMaj	16	TH	Starch	-29.011	11.07	-9.193	-9.126	0.0218	2.41
D07	PlaMed	16	SH	Glucose	-29.755	6.91	-10.904	-10.837	0.0207	1.43
D07	PlaMed	16	SH	Fructose	-29.755	1.50	-5.738	-5.671	0.0263	0.40
D07	PlaMed	16	SH	Sucrose	-29.755	4.10	-2.679	-2.612	0.0297	1.22
D07	PlaMed	16	SH	RFO	-29.755	5.35	-7.400	-7.333	0.0245	1.31
D07	PlaMed	16	SH	Starch	-29.755	10.15	-8.517	-8.450	0.0233	2.37
D08	AntOdo	4	GR	Glucose	-28.628	15.64	-3.561	-3.514	0.0275	4.30
D08	AntOdo	4	GR	Fructose	-28.628	2.85	-8.237	-8.190	0.0224	0.64
D08	AntOdo	4	GR	Sucrose	-28.628	43.44	-3.790	-3.744	0.0272	11.82
D08	AntOdo	4	GR	RFO	-28.628	114.74	-9.328	-9.281	0.0212	24.28
D08	AntOdo	4	GR	Starch	-28.628	7.84	-13.355	-13.309	0.0168	1.31
D08	PruVul	4	SH	Glucose	-29.899	9.04	-6.981	-6.935	0.0251	2.27
D08	PruVul	4	SH	Fructose	-29.899	NA	NA	NA	NA	NA
D08	PruVul	4	SH	Sucrose	-29.899	13.78	-3.157	-3.110	0.0293	4.04
D08	PruVul	4	SH	RFO	-29.899	29.91	-3.775	-3.729	0.0286	8.56
D08	PruVul	4	SH	Starch	-29.899	10.95	-12.902	-12.856	0.0186	2.04
D09	BelPer	16	SH	Glucose	-30.689	13.21	-15.129	-14.221	0.0180	2.38
D09	BelPer	16	SH	Fructose	-30.689	10.80	-13.040	-12.132	0.0203	2.19
D09	BelPer	16	SH	Sucrose	-30.689	13.01	-8.288	-7.381	0.0255	3.32
D09	BelPer	16	SH	RFO	-30.689	52.31	-12.705	-11.798	0.0207	10.81
D09	BelPer	16	SH	Starch	-30.689	16.53	-9.482	-8.575	0.0242	4.00
D09	CreBie	16	TH	Glucose	-29.634	13.67	-10.089	-9.181	0.0224	3.06
D09	CreBie	16	TH	Fructose	-29.634	21.81	-11.070	-10.163	0.0213	4.64
D09	CreBie	16	TH	Sucrose	-29.634	24.59	-5.245	-4.338	0.0277	6.80
D09	CreBie	16	TH	RFO	-29.634	17.75	-7.429	-6.521	0.0253	4.49
D09	CreBie	16	TH	Starch	-29.634	10.73	-12.121	-11.214	0.0202	2.16
D09	VerCha	16	SH	Glucose	-29.346	29.08	-18.935	-18.028	0.0124	3.60
D09	VerCha	16	SH	Fructose	-29.346	3.04	-14.316	-13.408	0.0174	0.53
D09	VerCha	16	SH	Sucrose	-29.346	5.30	-18.249	-17.342	0.0131	0.70
D09	VerCha	16	SH	RFO	-29.346	22.32	-19.443	-18.536	0.0118	2.64
D09	VerCha	16	SH	Starch	-29.346	9.47	-13.821	-12.914	0.0180	1.70
D10	PlaMed	4	SH	Glucose	-29.111	9.71	-7.313	-7.349	0.0238	2.31
D10	PlaMed	4	SH	Fructose	-29.111	3.88	-2.222	-2.258	0.0294	1.14
D10	PlaMed	4	SH	Sucrose	-29.111	4.19	-3.680	-3.716	0.0278	1.16

S8 Table continued

Dome	Species	SR	FG	NSC	$\delta^{13}C$ background (‰)	C (µg C mg_{dw}^{-1})	$\delta^{13}C$ uncorr (‰)	$\delta^{13}C$ corr (‰)	^{13}C atom% excess	^{13}C abundance (ng ^{13}C mg_{dw}^{-1})
D10	PlaMed	4	SH	RFO	-29.111	5.00	-3.295	-3.331	0.0282	1.41
D10	PlaMed	4	SH	Starch	-29.111	10.05	-6.368	-6.404	0.0248	2.50
D10	VicCra	4	LE	Glucose	-28.847	7.98	-4.152	-4.188	0.0270	2.15
D10	VicCra	4	LE	Fructose	-28.847	7.58	-2.314	-2.350	0.0290	2.20
D10	VicCra	4	LE	Sucrose	-28.847	23.15	-1.860	-1.896	0.0295	6.82
D10	VicCra	4	LE	RFO	-28.847	11.99	-1.360	-1.396	0.0300	3.60
D10	VicCra	4	LE	Starch	-28.847	12.15	-9.179	-9.215	0.0215	2.61
D11	AjuRep	16	SH	Glucose	-28.407	32.70	-21.250	-20.918	0.0082	2.68
D11	AjuRep	16	SH	Fructose	-28.407	2.60	-9.786	-9.453	0.0207	0.54
D11	AjuRep	16	SH	Sucrose	-28.407	14.66	-4.572	-4.240	0.0264	3.87
D11	AjuRep	16	SH	RFO	-28.407	89.78	-9.603	-9.270	0.0209	18.79
D11	AjuRep	16	SH	Starch	-28.407	9.59	-11.380	-11.047	0.0190	1.82
D11	ArrEla	16	GR	Glucose	-30.044	10.26	-12.066	-11.733	0.0200	2.06
D11	ArrEla	16	GR	Fructose	-30.044	2.69	-5.620	-5.287	0.0271	0.73
D11	ArrEla	16	GR	Sucrose	-30.044	51.65	-3.770	-3.438	0.0291	15.03
D11	ArrEla	16	GR	RFO	-30.044	19.31	-4.376	-4.043	0.0284	5.49
D11	ArrEla	16	GR	Starch	-30.044	6.74	-6.126	-5.793	0.0265	1.79
D11	FesPra	16	GR	Glucose	-28.796	5.61	-11.883	-11.550	0.0189	1.06
D11	FesPra	16	GR	Fructose	-28.796	3.21	-8.933	-8.600	0.0221	0.71
D11	FesPra	16	GR	Sucrose	-28.796	26.37	-5.561	-5.229	0.0258	6.80
D11	FesPra	16	GR	RFO	-28.796	39.59	-13.765	-13.433	0.0168	6.65
D11	FesPra	16	GR	Starch	-28.796	7.88	-7.552	-7.219	0.0236	1.86
D11	GleHed	16	SH	Glucose	-30.504	31.69	-19.275	-18.943	0.0126	4.01
D11	GleHed	16	SH	Fructose	-30.504	4.04	-8.942	-8.609	0.0240	0.97
D11	GleHed	16	SH	Sucrose	-30.504	3.84	-12.948	-12.616	0.0196	0.75
D11	GleHed	16	SH	RFO	-30.504	34.48	-10.461	-10.128	0.0223	7.69
D11	GleHed	16	SH	Starch	-30.504	13.31	-18.891	-18.558	0.0131	1.74
D11	HelPub	16	GR	Glucose	-29.404	11.50	-15.421	-15.088	0.0157	1.80
D11	HelPub	16	GR	Fructose	-29.404	1.85	-7.853	-7.520	0.0239	0.44
D11	HelPub	16	GR	Sucrose	-29.404	33.76	-3.508	-3.175	0.0287	9.69
D11	HelPub	16	GR	RFO	-29.404	46.71	-7.874	-7.541	0.0239	11.17
D11	HelPub	16	GR	Starch	-29.404	3.75	-6.307	-5.974	0.0256	0.96
D11	MedVar	16	LE	Glucose	-31.208	9.08	-9.786	-9.453	0.0238	2.16
D11	MedVar	16	LE	Fructose	-31.208	5.40	-5.880	-5.547	0.0281	1.52

S8 Table continued

Dome	Species	SR	FG	NSC	$\delta^{13}C$ background (‰)	C (µg C mg_{dw}^{-1})	$\delta^{13}C$ uncorr (‰)	$\delta^{13}C$ corr (‰)	^{13}C atom% excess	^{13}C abundance (ng ^{13}C mg_{dw}^{-1})
D11	MedVar	16	LE	Sucrose	-31.208	21.85	-4.685	-4.353	0.0294	6.42
D11	MedVar	16	LE	RFO	-31.208	16.24	-4.274	-3.942	0.0298	4.84
D11	MedVar	16	LE	Starch	-31.208	12.00	-11.689	-11.356	0.0217	2.61
D11	VicCra	16	LE	Glucose	-29.872	16.40	-6.316	-5.983	0.0261	4.29
D11	VicCra	16	LE	Fructose	-29.872	14.76	-2.462	-2.130	0.0303	4.48
D11	VicCra	16	LE	Sucrose	-29.872	18.06	-1.596	-1.263	0.0313	5.65
D11	VicCra	16	LE	RFO	-29.872	4.84	-2.485	-2.153	0.0303	1.47
D11	VicCra	16	LE	Starch	-29.872	12.81	-8.693	-8.361	0.0235	3.01
D12	MedVar	4	LE	Glucose	-30.002	8.84	-6.125	-5.397	0.0269	2.38
D12	MedVar	4	LE	Fructose	-30.002	7.38	-4.002	-3.274	0.0292	2.16
D12	MedVar	4	LE	Sucrose	-30.002	32.40	-1.872	-1.144	0.0316	10.23
D12	MedVar	4	LE	RFO	-30.002	19.84	-2.259	-1.531	0.0311	6.18
D12	MedVar	4	LE	Starch	-30.002	12.19	-9.621	-8.893	0.0231	2.82
D12	TriFla	4	GR	Glucose	-29.840	5.72	-6.335	-5.606	0.0265	1.52
D12	TriFla	4	GR	Fructose	-29.840	3.52	-4.338	-3.609	0.0287	1.01
D12	TriFla	4	GR	Sucrose	-29.840	62.54	-3.135	-2.407	0.0300	18.77
D12	TriFla	4	GR	RFO	-29.840	7.83	-3.147	-2.419	0.0300	2.35
D12	TriFla	4	GR	Starch	-29.840	9.66	-5.424	-4.696	0.0275	2.66
